# Case Report: Integrated genomic and immunological assays identify non-coding *CFB* variants in pneumococcal meningoencephalitis

**DOI:** 10.3389/fimmu.2026.1803307

**Published:** 2026-06-16

**Authors:** J. Barbieur, E. D’haenens, T. Jarayseh, L. Hoste, J. Smet, S. Lambrecht, E. Schiettecatte, P. Schelstraete, M. De Bruyne, F. Haerynck, S. J. Tavernier

**Affiliations:** 1 Primary Immune Deficiency Research Lab (PIRL), Department of Internal Medicine and Pediatrics, Ghent University, Ghent, Belgium; 2 Center for Primary Immunodeficiency Ghent, Jeffrey Modell Diagnosis and Research Center, ERN-RITA reference center, Ghent University Hospital, Ghent, Belgium; 3 Department of Pediatric Pulmonology, Immunology and Infectiology, Ghent University Hospital, Ghent, Belgium; 4 Center for Medical Genetics, Ghent University Hospital, Ghent, Belgium; 5 Department of Biomedical Molecular Biology, Ghent University, Ghent, Belgium; 6 Department of Pediatrics, Ghent University Hospital, Ghent, Belgium; 7 Department of Immunology, Laboratoire Hospitalier Universitaire de Bruxelles - Universitair Laboratorium Brussel (LHUB-ULB), Université Libre de Bruxelles (ULB), Brussels, Belgium; 8 Immunology Department, University Hospital Brugmann, Université Libre de Bruxelles (ULB), Brussels, Belgium; 9 Department of Laboratory Medicine, Ghent University Hospital, Ghent, Belgium; 10 Department of Radiology and Nuclear Medicine, Ghent University Hospital, Ghent, Belgium

**Keywords:** case report, FB deficiency, pneumococcal meningoencephalitis, rescue assays, Transcriptomics

## Abstract

Inborn defects of the alternative pathway (AP) of the complement system have revealed its essential role in host defense against invasive infections caused by encapsulated bacteria. Biallelic pathogenic variants in the *CFB* gene cause complement factor B (FB) deficiency, an exceptionally rare inborn error of immunity (IEI), with only three cases reported to date. Here, we describe a fourth case of autosomal recessive FB deficiency. An 8-month-old boy with no significant medical history presented with severe pneumococcal bacteremia with clinical and radiological features of meningoencephalitis. We performed comprehensive clinical, immunological, genetic, and functional analyses. Complement studies revealed absent AP activity with preserved classical and lectin pathway function. Quantification of individual complement components showed markedly reduced FB levels with no rise of FBb and normal concentrations of properdin, factor D (FD), factor H (FH), factor I (FI), C3 and C3d, consistent with a primary defect in FB production. Targeted genetic testing initially identified a single splice-site *CFB* variant (c.898-2A>C), but the profound alterations in the biochemical phenotype suggested a missed second *CFB* variant. Additional in-depth analyses using whole-exome sequencing revealed the presence of an additional non-canonical splice-site *CFB* variant (c.1168 + 10G>T). RNA sequencing of patient-derived dermal fibroblasts followed by Sanger sequencing demonstrated that both variants caused aberrant splicing, including exon skipping and cryptic splice-site activation. Segregation analyses confirmed compound heterozygosity in the proband, while the heterozygous carrier of the c.1168 + 10G>T variant showed reduced FB levels but preserved AP activity. Rescue assays functionally validated the molecular diagnosis, as AP activity could not be restored by mixing with FB-deficient serum but was rescued by supplementation with recombinant FB. This case expands the genetic spectrum of FB deficiency to include non-coding *CFB* variants and highlights the critical value of integrating in-depth functional immunological assays with advanced genomic and transcriptomic analyses to establish a definitive diagnosis in rare cases of IEI.

## Introduction

As an integral component of humoral innate immunity, the complement system provides critical protection against invasive bacterial infections. Beyond its role in host defense, it also contributes to immune homeostasis through the direct interactions with components of the adaptive immune response and clearance of apoptotic debris and immune complexes (1). Comprising more than 50 proteins in a zymogen cascade, the complement system rapidly amplifies immune responses following activation of the classical pathway, the lectin pathway, or the alternative pathway. Components of the alternative pathway, such as complement factor B (FB), D (FD) and properdin contribute to full activation of the complement system following initiation via the classical and lectin pathway. Collectively, these pathways converge on the cleavage of C3 by the C3 convertase, promoting clearance of pathogens through opsonization and recruitment of immune cells. Subsequent incorporation of C3b into the C3 convertase leads to the formation of the C5 convertase, which initiates the terminal pathway, culminating in membrane attack complex assembly and cytolysis of target cells (1).

Consequently, inborn errors of the complement system result in an increased susceptibility to encapsulated bacterial infections but also predispose to immune-mediated diseases, including systemic lupus erythematosus and atypical hemolytic uremic syndrome (1). Complement deficiencies are rare, representing only 4.8% of all inborn errors of immunity (IEI) reported in the European Society for Immunodeficiencies (ESID) registry (2). Among these, FB deficiency is exceptionally rare, with only three cases reported to date, all presenting with recurrent infections by *S. pneumoniae* and *N. meningitidis* (3–5). Here, we describe a fourth case of this extremely rare autosomal recessive IEI.

## Case presentation

**Figure 1:**
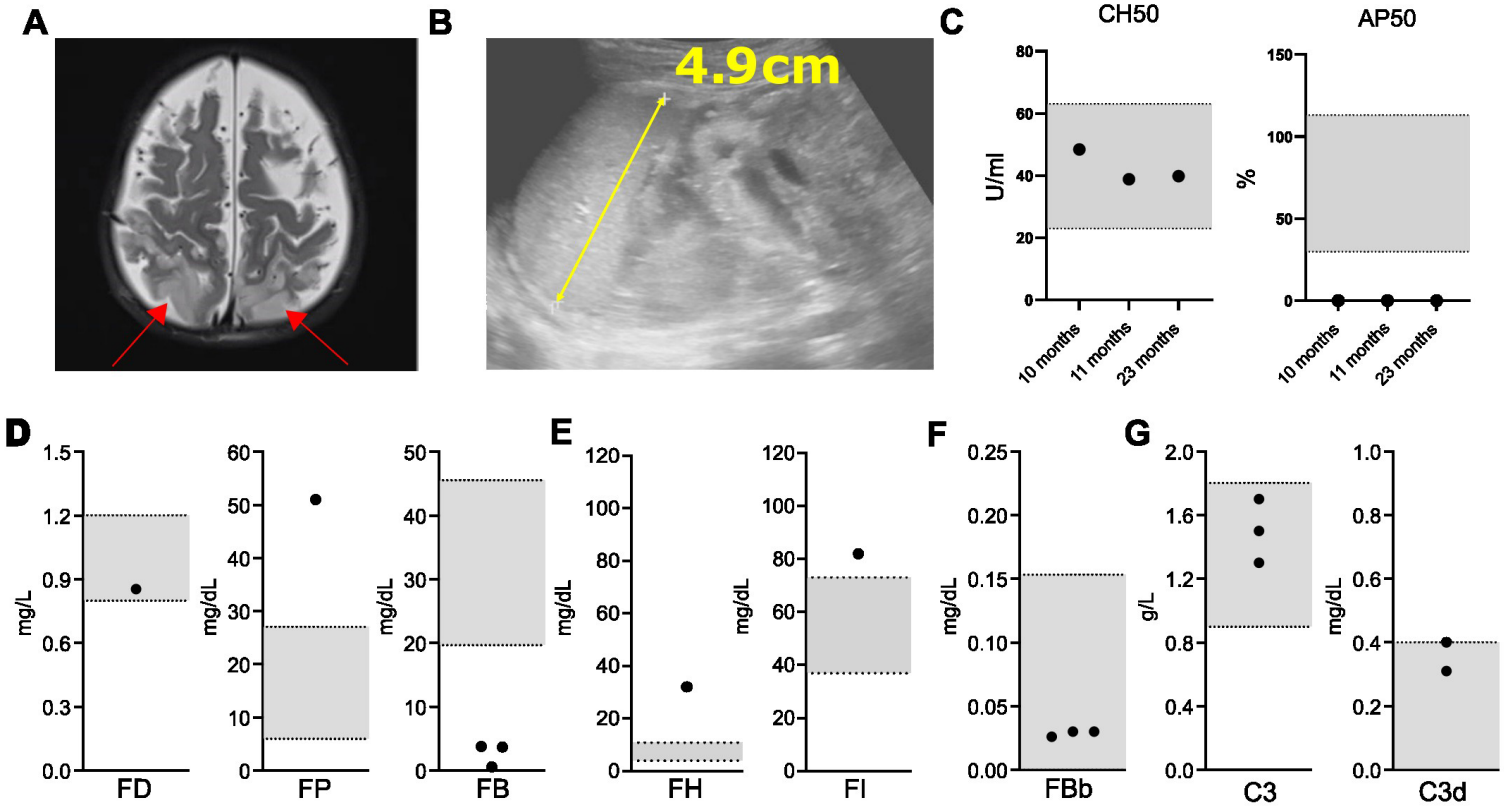
Immunological work-up of the proband **(A)** Axial T2-weighted MRI image of the proband demonstrating multifocal cortical hyperintensity, most pronounced in the high posterior parietal regions (arrows), corresponding to areas of extensive encephalitis. **(B)** Abdominal ultrasound showing normal structure and size of the spleen. Line indicates maximal spleen size. **(C)** Bargraph depicting functional complement testing in serum of the proband, assessing classical pathway (CH50; U/mL; n=3) and alternative pathway (AP50; %; n=3) activity. Each dot represents an independent timepoint. **(D)** Bargraph quantifying serum concentration of factor D (FD; mg/L), properdin (FP; mg/dL) and factor B (FB; mg/dL). **(E)** Bargraph showing serum concentration of regulatory factors H (FH; mg/dL) and factor I (FI; mg/dL). **(F)** Bargraph showing serum concentration of factor Bb (FBb; mg/dL; n=3). **(G)** Bargraph showing serum concentration of C3 (g/L; n=3) and its degradation product C3d (mg/dL; n=2). Normal reference values are indicated in grey.

An 8-month-old boy presented to the emergency department with a four-day history of fever and irritability, accompanied by hemodynamic instability compatible with sepsis. He had no significant medical history and was vaccinated according to the Belgian national vaccination schedule (including two doses of a 13-valent pneumococcal conjugate vaccine and three doses of a *H. influenzae* type b vaccine). He is the first child of non-consanguineous parents. The combination of fever and irritability raised clinical suspicion of bacterial meningitis, and treatment with cefotaxime and dexamethasone (0.15 mg/kg) was promptly initiated. Blood sampling at admission showed high inflammatory markers with a C-reactive protein of 120mg/L (*<5 mg/L*), lymphopenia (1260/µL, reference values *4000-10500/µL*) and neutrophilia (12340/µL, reference values *1500-8500/µL*). Lumbar puncture for cerebrospinal fluid cultures was deferred due to the patient’s hemodynamic instability. Blood cultures were positive for *S. pneumoniae* type 35F (not included in the 13-valent conjugate vaccine). Brain MRI revealed abnormalities compatible with meningoencephalitis (**Figure 1A**). Despite timely microbial therapy and intensive care unit admission, the pneumococcal meningoencephalitis led to long-term neurological sequelae, including unilateral neurosensorial hearing loss and epilepsy. The patient, currently 4 years of age, is in good overall health. He is currently reviewed at six-monthly intervals in a specialized immunology outpatient clinic. Ongoing management includes daily prophylactic oral pheneticillin administration. He has been provided with an emergency alert letter detailing his complement deficiency and the associated risk of invasive infections with encapsulated bacteria.

Immunization was comprehensively optimized, prioritizing vaccines against encapsulated bacteria. In addition to the conjugated meningococcal A/C/W/Y vaccine included in the national immunization schedule, he received conjugated meningococcal B vaccination. Booster doses for meningococcal serogroups A, C, W, Y and B are scheduled every 3–5 years. Beyond 13-valent conjugated pneumococcal vaccines, he received an unconjugated 23-valent polysaccharide pneumococcal vaccine at age 2 years with good antibody response. He recently received one dose of 20-valent conjugated pneumococcal vaccine. Vaccination with live-attenuated varicella zoster virus was performed. Annual influenza vaccination is strongly advised both for the patient and his close contacts, as influenza infection in patients with complement disorders may predispose to secondary encapsulated bacterial infections.

## Results

Immunological work-up showed normal spleen structure on ultrasound (**Figure 1B**) and absence of Howell Jolly bodies. Complete blood count and differential were normal, as well as B and T lymphocyte populations and maturation. Immunoglobulin G (IgG), IgG2 and IgG3 subclasses, IgA and IgM concentrations were appropriate for age and an adequate antibody response to the unconjugated 23-valent pneumococcal polysaccharide vaccine was observed (for a detailed overview of clinical, immunological and genetic findings, see **Table 1**). Functional complement assays were performed. Classical pathway activation (CH50), using a liposome-based technique (iSYS, Immunodiagnostic Systems Ltd), was repeatedly within normal values (*23–63 U/mL*). The lectin pathway activation was similarly normal (*data not shown*). The alternative pathway was assessed using a pathway-specific ELISA kit (AP50, WIESLAB^®^ Complement Alternative Pathway, SVAR life sciences) and revealed repeatedly absent AP50 in the proband (reference values *30-113%*) (**Figure 1C**).

**Table 1:** Overview of published complement factor B deficiency cases.

Article	Year of publication	Sex	Ethnicity	Infection				Lymphocyte subpopulations	IgG/A/M	Pneumococcal vaccine response	CH50	AP50	FB	Genetics
				Age	Diagnosis	Pathogens identified	Complications							
Slade C, Bosco J, Unglik G, Bleasel K, Nagel M, Winship I. Deficiency in complement factor B. N Engl J Med. 2013 Oct 24;369(17):1667-9. doi: 10.1056/NEJMc1306326. PMID: 24152280.	2013	Female	English-Scottish	2 yo	Peritonitis	Pneumococ, not further differentiated		Normal	Normal	NA	Normal	0% (*N >65%)*	Undetectable, <36g/L (*N 119-245g/L*)	*CFB* (NM_001710.5) p.(Gln256Ter/p.(Phe632CysfsTer8). c.766C>T/c.1894_1897delTTTG
				4 yo	Community-acquired pneumonia	NA								
				15 yo	Meningitis	Neisseria meningitidis, serogroup Y								
				30 yo	Pneumonia	Pneumococ, not further differentiated	Type 1 respiratory failure, unilateral empyema							
Gauthier A, Wagner E, Thibeault R, Lavoie A. A Novel Case of Complement Factor B Deficiency. J Clin Immunol. 2021 Jan;41(1):277-279. doi: 10.1007/s10875-020-00906-3. Epub 2020 Nov 9. PMID: 33165708.	2021	Male	French Canadian	4 months	Bacteremia	Pneumococ, not further differentiated		Normal	Normal	Normal	Normal	0% (*N 30-113%)*	144 mg/L (*N 173-453mg/L)*	*CFB* (NM_001710.5) p.(Ile647HisfsTer6) / p.(Gly396Arg). c.1938dup/c.1186G>A
				5 months	Perioribital cellulitis	NA								
				11 months	Meningitis	Pneumococ, not further differentiated								
				5 yo	Pneumonia	Streptococcus pneumoniae	Unilateral empyema							
				8 yo	Pneumonia	No bacteria identified	Septic shock, acute respiratory distress syndrome, thromboembolism right femorial artery, partial first toe amputation							
Bougeard C, Eskander E, Martins PV, Bitan J, Roquigny J, Kyheng C, Guiddir T, Frémeaux-Bacchi V, Gonnin C, ElSissy C. Meningococcal Serogroup Y Meningitis Reveals Inborn Factor B Deficiency. Eur J Immunol. 2026 Feb;56(2):e70148. doi: 10.1002/eji.70148. PMID: 41663882; PMCID: PMC12886615.	2026	Female	French	17 months	Pneumonia	Likely pneumococ		NA	NA	NA	Normal	<10% (*N 70-130%*)	0.132 g/L (*N 0.198-0.455 g/L*)	*CFB* (NM_001710.6) p.Gly396Arg/p.Gln713Arg). c.1186G>A/c.2138A>G
				8 yo	Appendicular peritonitis	NA								
				14 yo	Meningitis	Neisseria meningitidis, serogroup Y	Bacteremia, cerebral venous sinus thrombosis							
This article	2026	Male	Belgian	8 months	Meningoencephalitis	Streptococcus pneumoniae, type 35F	Unilateral neurosensorial hearing loss, epilepsy	Normal	Normal	Normal	Normal	0% (*N 30-113%*)	0.56-3.75 mg/dL *(N 19.7-45.5mg/dL*)	*CFB* (NM_001710.6) p.?/p.(Leu391Glnfs*47). c.898-2A>C / c.1168+10G>T

Abbreviations: T, Lymphocyte subpopulations; B and NK, cell populations; Ig, immunoglobulin; CH50, classical complement pathway activation; AP50, alternative complement pathway activation; FB, complement factor B level; yo, years old; NA, not available.

We quantified individual complement factors using specific human Properdin or FD ELISA kits (Sanbio^®^) and found high levels of properdin (51 mg/dL, reference values *6.0-27.0 mg/dL*), whereas FD concentration was within normal reference values (0.85 mg/dL, reference values *0.4-1.2 mg/dL*). In contrast, FB levels, measured by nephelometry (Trimero Diagnostics^®^), were markedly reduced across repeated measurements (0.56-3.75 mg/dL, reference values *19.7-45.5 mg/dL*, **Figure 1D**). The concentrations of the regulatory factors (Nephelometry, Binding Site^®^) FH (82mg/dL) and FI (32mg/dL) were above their respective reference ranges (*37–73 mg/dL* and *4.0-10.7 mg/dL*), rendering dysregulation of the alternative pathway due to a deficiency of these factors unlikely (**Figure 1E**). In line with these observations, the concentration of the factor B cleavage product FBb (Microvue^®^ ELISA) was below the limit of detection (*<0.153 mg/dL*) (**Figure 1F**). Furthermore, serum concentration of C3 (Turbidimetry, Binding Site^®^) and its degradation product C3d (Nephelometry, Dako^®^) were within the normal limits (*0.9-1.8 g/L* and <*0.4 mg/dL*, respectively, **Figure 1G**), arguing against ongoing consumption due to alternative pathway overactivation (6).

**Figure 2:**
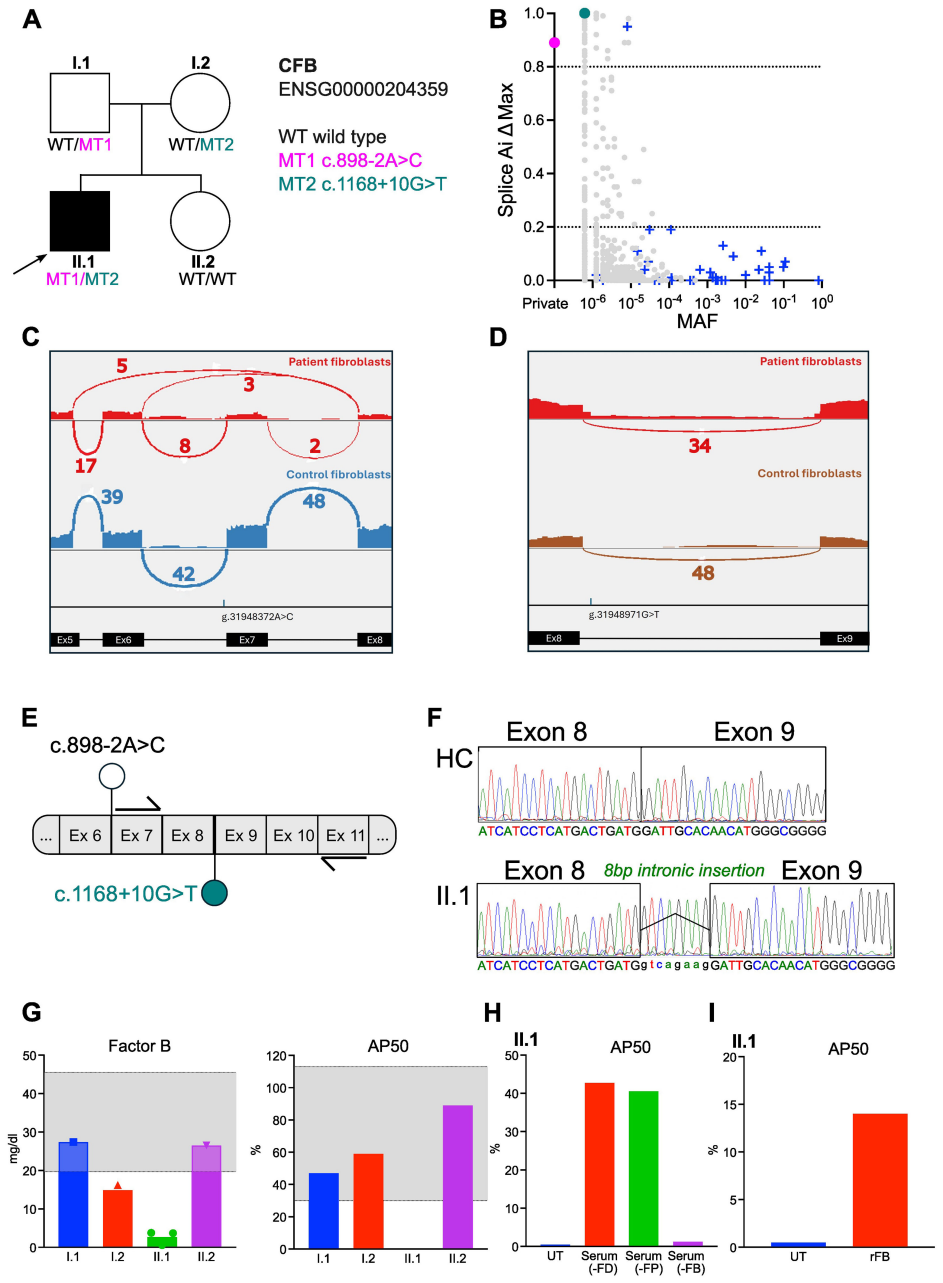
Genetic and functional work-up of proband and family members. **(A)** Pedigree of the family with allele segregation of *CFB* non-coding variants. The proband is marked with an arrow and genotype is indicated in the legend. **(B)** In silico splice prediction (Splice AI delta score) and minor allele frequency (MAF) of *CFB* variants found in the proband (MT1 in fuchsia, MT2 in teal), compared to heterozygous (grey dots) and homozygous (blue crosses) variants within ±50 base pairs of exon-intron boundaries, as reported in population database gnomAD v.4.1.0. **(C)** Sashimi plots depicting RNA sequencing junction reads for the c.898-2A>C *CFB* variant. The proband’s fibroblasts are shown in red, healthy control fibroblasts in blue, with maximum sequencing coverages of 20X and 60X respectively. Numbers represent sequencing read counts. **(D)** Sashimi plots depicting RNA sequencing junction reads for the c.1168 + 10G>T *CFB* variant. The proband’s fibroblasts are shown in red, healthy control fibroblasts in brown, with maximum sequencing coverages of 20X and 60X, respectively. Numbers represent sequencing read counts. **(E)** Schematic illustration of CFB variant locations and the primer pair (black arrows) used for PCR amplification of cDNA encompassing the c.1168 + 10G>T variant (green pin). The variant not included in Sanger sequencing is depicted by a white pin. **(F)** Electropherograms of cDNA extracted from dermal fibroblasts of the proband and a healthy control. Genomic localization on chromosome 6 (GRCh38) is given. **(G)** Bargraph showing concentration of FB and AP50 in respectively plasma and serum of the proband (II.1) and family members I.1, I.2 and II.2. Each dot represents an independent timepoint (1 for I.1, 2 for I.2, 3 for II.1 and 1 for II.2). Normal reference values are indicated in grey. **(H)** Hemolytic rescue assay assessing AP50 activity of serum of the proband complemented with serum depleted of factor D(FD, red), properdin (FP, green) or factor B (FB, purple) compared to the untreated serum of the proband (UT, blue). This figure represents data of one independent experiment. **(I)** Hemolytic rescue assay assessing AP50 activity of untreated serum of the proband (UT, blue) and complemented with recombinant factor B (rFB, red).

Targeted sequencing of a panel of genes encoding complement factors identified an intronic variant in *CFB* (NM_001710.6, c.898-2A>C). Given the profound biochemical defect, a missed second variant was suspected and whole exome sequencing was performed, identifying an additional non-coding variant in *CFB* (c.1168 + 10G>T). No alternate candidate variants in other complement factors were identified. Segregation analysis demonstrated paternal inheritance of the c.898-2A>C and maternal inheritance of the c.1168 + 10G>T, confirming compound heterozygosity in the proband. The patient’s sister did not carry either of *CFB* variants (**Figure 2A**). The c.898-2A>C *CFB* variant (located in intron 6) is reported in a heterozygous state at a low minor allele frequency (MAF: 0.0000681%) in the reference population cohorts such as the Genome Aggregation Database (gnomAD) and without reported homozygotes. The variant disrupts the canonical splice acceptor site and has strong predictions to alter splicing by multiple in silico tools, including SpliceAI and Pangolin (Delta scores: acceptor loss 0.89; splice loss 0.78; **Figure 2B**). The c.898-2A>C variant has already been reported in ClinVar as likely pathogenic (ID:3066149). In case of the second *CFB* variant, c.1168 + 10G>T, located in intron 8, only one allele has been reported in gnomAD, accounting for a MAF of 0.000062%. This variant is not reported in ClinVar. Although this variant is a non-canonical splice-site variant, in silico tools (SpliceAI and Pangolin) predict aberrant splicing (Delta scores: donor gain 1.0, splice gain 0.87; **Figure 2B**). To date, only three additional cases with biallelic loss-of-function (LOF) *CFB* variants underlying FB deficiency have been reported (**Table 1**, 3,4,5). Homozygous LOF mutations such as nonsense and frameshift variants are absent in gnomAD, although 1 homozygous intronic variant with a strong predicted splicing impact was identified (**Figure 2B**). Taken together, these data support the identified intronic *CFB* variants as the likely molecular cause of FB deficiency in the proband.

Hepatocytes are the primary cellular source of FB, precluding the use of leukocytes for functional analyses (7). To functionally validate the impact of both *CFB* variants on mRNA maturation, we performed RNA sequencing (RNA-seq, Stranded mRNA Prep, Ligation kit, Illumina^®^) on patient-derived dermal fibroblasts (8). Prior to cell lysis and RNA extraction (RSC simplyRNA Tissue Kit, Maxwell^®^), fibroblasts were cultured with or without puromycin to inhibit nonsense-mediated mRNA decay. RNA-seq analysis revealed alternative junction reads spanning exon 5–8 and exon 6-8, indicating aberrant splicing associated with the c.898-2A>C variant, and skipping of exon 6 and exon 7 (**Figure 2C**). In contrast, for the c.1168 + 10G>T variant, no alternative splice junctions were observed but in-depth analysis of the RNA-seq data revealed partial retention of intron 8 (**Figure 2D**). To validate these RNA-seq findings, Sanger sequencing of amplified cDNA, spanning exons 7–11, was performed (**Figure 2E**) and confirmed that the c.1168 + 10G>T variant activates a cryptic splice-site, resulting in the retention of 8 base pairs of intron 8. This aberrant splicing event is predicted to cause a frameshift and introduce a premature termination codon (p.(Leu391Glnfs*47)) (**Figure 2F**).

A detailed functional work-up of the family members of the proband was conducted to further validate the effects of both *CFB* variants on FB levels and complement activation. As expected, the sibling, carrying biallelic wild type *CFB* alleles, presented with normal alternative pathway activation and FB levels within reference values. The father of the proband, heterozygous for *CFB* c.898-2A>C, displayed both normal AP50 (47%) and FB concentration (27.4 mg/dL). In contrast, analysis of the plasma of the mother, carrying the c.1168 + 10G>T non-coding variant, revealed a repeatedly decreased FB concentration (13.5-16.2 mg/dL, reference values *19.7-45.5 mg/dL*). As expected for heterozygous carriership, AP50 in the mother remained within the normal range (59%, **Figure 2G**). Finally, to functionally confirm that the observed FB defect was responsible for the impaired alternative pathway activation, we performed rescue/complementation assays. Alternative pathway activity was restored upon mixing the proband’s serum with properdin-deficient or factor D–deficient sera. In contrast, AP50 remained severely reduced (1.2%) when the proband’s serum was mixed with factor B–deficient serum (**Figure 2H**). Repletion of the proband’s serum with commercially available recombinant FB restored AP50 to 14% (**Figure 2H**), functionally validating the molecular diagnosis of autosomal recessive factor B deficiency.

## Discussion

In 2013, Slade et al. described a 32-year-old woman with recurrent pneumococcal and meningococcal infections, with disease onset at the age of 2 years. Functional testing revealed an inactive alternative pathway and undetectable complement factor B (FB). Targeted sequencing of the *CFB* gene (NM_001710.5) identified compound heterozygous truncating variants (p.(Gln256Ter) and p.(Phe632CysfsTer8)). A second case was reported in 2021 by Gauthier et al., describing an 8-year-old boy with recurrent invasive pneumococcal infections harboring a frameshift variant, p.(Ile647HisfsTer6), and a missense variant, p.(Gly396Arg). More recently, Bougeard et al. described a third case of factor B deficiency in a 14-year-old girl who presented with probable pneumococcal pneumonia, appendicular peritonitis, and severe meningococcal serogroup Y meningitis. Targeted sequencing identified two missense variants in *CFB*, p.(Gly396Arg) and p.(Gln713Arg) (5). In the cases presented by Gauthier et al. and Bougeard et al, factor B levels were nearly normal despite complete functional protein impairment and strongly impaired alternative pathway activation, indicating a qualitative rather than a quantitative defect (4, 5). In all described cases, patients received vaccination against *S. pneumoniae* and *N. meningitidis*, as well as antibiotic prophylaxis with oracillin or amoxicillin. No additional severe infections or other immune-related conditions were observed (3–5). Here, we report the fourth case of FB deficiency, presenting with an invasive infection by *S. pneumoniae* at the age of 8 months, neurological sequelae and a quantitative FB deficiency, expanding the clinical and genetic spectrum of FB deficiency to include biallelic non-coding *CFB* variants as a novel disease mechanism (**Table 1**).

The clinical and genetic diagnostic challenges illustrated by this case are emblematic of the diagnostic odyssey faced by patients with inborn errors of immunity (IEI) and their caregivers. This emphasizes the importance of expert clinical recognition to timely guide integrated, comprehensive complement testing, prioritizing the functional analysis of the classical pathway (CH50) and the alternative pathway (AP50), in combination with the dosing of specific complement factor levels and advanced genetic diagnostics (9). Importantly, deficiencies of the alternative complement pathway are likely underdiagnosed, in part because the use of hemolytic assays such as AP50 or the ELISA−based functional alternatives are confined to specialized laboratories and not routinely performed by clinicians. Functional testing of the alternative pathway is especially important in the case of qualitative factor B deficiencies where missense variants preserve near-normal antigenic factor B levels (**Table 1****, 4, 5**). As such, FB deficiency as an underlying cause of invasive infections with encapsulated bacteria may be underrecognized, and AP50 testing should be systematically incorporated into the diagnostic evaluation of suspected cases.

A systematic review of children with invasive pneumococcal disease identified age (>2 years), recurrence, and site of infection (pneumonia and meningitis) as key indicators of an underlying IEI (10). In line with this, the widely used primary immunodeficiency warning signs propose recurrent invasive infections as a red flag for an unrecognized IEI. However, these guidelines no longer reflect the breadth of clinical presentations, as up to 30% of patients with IEI do not meet the warning signs (11). The presentation of a single invasive infection with a pneumococcal serotype not currently covered by conjugate vaccines (35F) at a young age in our proband exemplifies these shortcomings. We advocate a more nuanced approach in which clinical acumen and a low threshold for first line functional complement testing prompt additional molecular investigation. This is particularly important given the extreme rarity of FB deficiency (four cases including this report) and our limited understanding of the phenotypic spectrum associated with this disorder. Notably, in contrast to previously reported cases of FB deficiency (**Table 1**), our patient did not experience recurrent invasive infections to date. This difference may be attributable to early diagnosis and/or the timely initiation of prophylactic antibiotic therapy.

Finally, this case highlights the potential limitations of a “genetics-first” approach in the field of inborn errors of immunity. The initial targeted molecular screening of the complement pathway only identified the c.898-2A>C *CFB* variant. Parallel comprehensive functional complement testing, however, revealed absent AP50 activity and persistently reduced FB levels, confirming the clinical suspicion of missing heritability and prompting further familial complement work-up, guiding additional genetic investigation and identifying the c.1168 + 10G>T variant. This report exemplifies how integration of detailed knowledge of the human immune system and in-depth immune profiling technologies with genomic diagnostic are prerequisites for characterizing and validating novel IEI genes and variants.

## Data Availability

The data of this patient is available upon request to the corresponding authors.
